# Evaluation of kidney motion and target localization in abdominal SBRT patients

**DOI:** 10.1120/jacmp.v17i6.6406

**Published:** 2016-11-08

**Authors:** Marcus Sonier, William Chu, Nafisha Lalani, Darby Erler, Patrick Cheung, Renee Korol

**Affiliations:** ^1^ Medical Physics Sunnybrook Odette Cancer Centre Toronto ON Canada; ^2^ Department of Radiation Oncology University of Toronto Toronto ON Canada

**Keywords:** renal cell carcinoma, stereotactic body radiation therapy (SBRT), motion management, Elekta BodyFIX, CBCT, setup error

## Abstract

The purpose of this study was to evaluate bilateral kidney and target translational/rotational intrafraction motion during stereotactic body radiation therapy treatment delivery of primary renal cell carcinoma and oligometastatic adrenal lesions for patients immobilized in the Elekta BodyFIX system. Bilateral kidney motion was assessed at midplane for 30 patients immobilized in a full‐body dual‐vacuum‐cushion system with two patients immobilized via abdominal compression. Intrafraction motion was assessed for 15 patients using kilovoltage cone‐beam computed tomography (kV‐CBCT) datasets (n=151) correlated to the planning CT. Patient positioning was corrected for translational and rotational misalignments using a robotic couch in six degrees of freedom if setup errors exceeded 1 mm and 1°. Absolute bilateral kidney motion between inhale and exhale 4D CT imaging phases for left–right (LR), superior–inferior (SI), and anterior–posterior (AP) directions was 1.51±1.00mm,8.10±4.33mm, and 3.08±2.11mm, respectively. Residual setup error determined across CBCT type (pretreatment, intrafraction, and post‐treatment) for x (LR), y (SI), and z (AP) translations was 0.63±0.74mm,1.08±1.38mm, and 0.70±1.00mm; while for x (pitch), y (roll), and z (yaw) rotations was 0.24±0.39°,0.19±0.34°, and 0.26±0.43°, respectively. Targets were localized to within 2.1 mm and 0.8° 95% of the time. The frequency of misalignments in the y direction was significant (p<0.05) when compared to the x and z directions with no significant difference in translations between IMRT and VMAT. This technique is robust using BodyFIX for patient immobilization and reproducible localization of kidney and adrenal targets and daily CBCT image guidance for correction of positional errors to maintain treatment accuracy.

PACS number(s): 87.55.‐x, 87.56.‐v, 87.56.Da

## I. INTRODUCTION

Motion management is an important consideration during radiotherapy treatment, particularly in the case of stereotactic body radiation therapy (SBRT) where a high dose per fraction is utilized. Organ motion due to breathing during treatment delivery can result in an organ at risk (OAR) entering the treatment beam and subsequently exceed the tolerance dose while the target may move outside of the treatment beam, restricting the dose it receives to less than the intended prescription dose. Various CT and MR studies report kidney motion due to shallow respiration to occur in a complex pattern with the largest movement in the superior–inferior direction, an average of 6.7–19.0 mm.^1^, ^2^, ^3^, ^4^, ^5^, ^6^, ^7^ In order to limit OAR and target motion, a variety of immobilization techniques are available and explained in detail by the report of AAPM Task Group 76, which contains the recommendation that in the cases where a ≥5mm range of motion is observed, respiratory management techniques should be employed.^1^, ^2^ In addition to internal motion, patient setup uncertainty and intrafraction movement during radiotherapy treatment limits the accuracy and effectiveness of the delivered treatment plan, particularly in the case of SBRT where small PTV margins are utilized and high doses result in longer treatment times. A direct result of these small margins in conjunction with high doses necessitates correcting rotational misalignments in order to achieve increased target localization accuracy and acceptable setup uncertainties reflective of the PTV margins applied.^(8)^ Thus, the specific aim of this study is to assess maximum kidney motion from 4D CT datasets and intrafraction target motion using kilovoltage cone‐beam computed tomography (kV‐CBCT) for patients immobilized in the BodyFIX system (Elekta AB, Stockholm, Sweden) with patient positioning corrected for both translational and rotational errors using a robotic couch in six degrees of freedom.

## II. MATERIALS AND METHODS

This study was approved by the institutional ethics committee. Intrafraction motion was retrospectively evaluated by analyzing 4D CT datasets and kV‐CBCT registration records.

Bilateral kidney motion was assessed for 30 patients (a combination of liver and kidney/adrenal SBRT patients) who were immobilized in the BodyFIX system (a molded full‐body vacuum bag into which the patient is vacuum sealed via a plastic sheet placed on the patient's anterior surface) with two patients immobilized in the BlueBAG (Elekta) with abdominal compression. Absolute motion was measured at midplane in the left–right, superior–inferior, and anterior–posterior directions using the inhale and exhale phases of the helical 4D CT acquired at simulation.

Intrafraction motion was then determined from the kV‐CBCT setup records of residual error for a subset of 15 patients with renal cell carcinoma or adrenal metastases treated with either VMAT (n=10) or IMRT (n=5). The planning CT and CBCT were registered using an automatic gray value match to the PTV and adjacent kidney with manual fine‐tuning adjustments made to ensure that both the target volume and bony anatomy between the image sets were correlated. CBCT registrations were reviewed by a radiation oncologist. For all cases, patient positioning was corrected using a robotic couch in six degrees of freedom if the setup errors exceeded a threshold of 1 mm and 1°. CBCTs were acquired and shifts applied if setup errors exceeded the acceptable threshold following the initial setup registration, pretreatment scan, and intrafraction scan if IMRT was used due to long treatment times. A post‐treatment CBCT scan was acquired to verify the patient's final position. Translational and rotational errors were recorded for each CBCT registration. In total, 151 CBCT records were included in the localization analysis.

Residual patient errors were analyzed using analysis of variance (ANOVA) to evaluate directional differences and, provided the ANOVA was significant (p<0.05), a post hoc analysis was used to evaluate pairwise comparisons. An unpaired *t*‐test was used to evaluate differences in patient positioning errors between IMRT and VMAT treatment groups. The 95% confidence interval (CI) was calculated for translations and rotations.

## III. RESULTS

**Figure 1:**
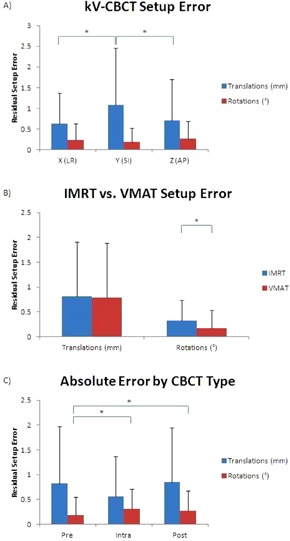
Summary of translational and rotational positional errors based on 151 image registrations. The absolute errors are represented as a mean and standard deviation. The data were separated as: (a) x (LR), y (SI), and z (AP); (b) IMRT and VMAT; and (c) pre‐, intra‐, and post‐treatment CBCT. The * indicates statistical significance between the respective results at p<0.05.

The evaluation of absolute bilateral kidney motion across the inhale and exhale 4D CT image acquisitions in the left–right, superior–inferior, and anterior–posterior directions represented as a mean and standard deviation showed breathing induced displacements of 1.51±1.00mm,8.10±4.33mm, and 3.08±2.11mm, respectively. Residual setup error, including intrafraction motion, determined by kV‐CBCT for x (left–right), y (superior–inferior), and z (anterior–posterior) translations was 0.63±0.74mm,1.08±1.38mm, and 0.70±1.00mm; while for x (pitch), y (roll), and z (yaw) rotations was 0.24±0.39°,0.19±0.34°, and 0.26±0.43°, respectively. The frequency of translational misalignments in the y direction was found to be statistically significant (p<0.001) when compared to the x and z directions while the rotational misalignments were statistically significant when considering intrafraction motion (p=0.027) as well as for IMRT vs. VMAT (p<0.001), as illustrated in Fig. 1. Table 1 displays the frequency of misalignments for both translations and rotations broken down by direction and CBCT type. The 95% CI showed an overall localization accuracy of 2.1 mm (1.5 mm, 2.7 mm, and 2.0 mm in the x, y, and z directions, respectively) and 0.8° over 15 patients using a correction threshold of 1 mm and 1°.

**Table 1:** Frequency of misalignments indicated by CBCT matching for all patients

		*Frequency (%)*	
		*Translations*	*Rotations*
	Pretreatment	27	7
X (LR/pitch)	Intrafraction	11	21
	Post‐treatment	25	7
	Pretreatment	41	4
Y (SI/roll)	Intrafraction	32	0
	Post‐treatment	41	6
	Pretreatment	26	13
Z (AP/yaw)	Intrafraction	21	7
	Post‐treatment	29	7

## IV. DISCUSSION

The assessment of bilateral kidney motion for patients immobilized in the dual‐vacuum‐cushion system show the largest deviation in the superior–inferior direction with a subset of patients displaying translations larger than 10 mm, while the left–right and anterior–posterior translations were also substantial, albeit consistently lower. These sizeable motions agree with existing data concerning kidney movement due to breathing motion and corroborate the choice to use an ITV for target localization.^(6,7,9–11)^ The residual setup error determined from the on‐line CBCT (n=151) alignment corresponds with the absolute motion of the kidneys where the greatest error in localization accuracy occurred in the y direction (superior–inferior) compared to the x (left–right) and z (anterior–posterior) directions, consistent with studies in the literature.^8^, ^12^ Despite patient immobilization in the dual‐vacuum‐cushion system this observation illustrates that the impact of breathing motion on target localization remains clinically significant and strategies to further reduce kidney motion will be investigated. Possible techniques involve breath‐hold techniques or respiration‐gated radiation therapy as a method to minimize the volume of tissue irradiated, geometrically sparing OARs.^(13)^ The main strength of this study is the assessment of both translational and rotational setup errors for kidney and adrenal SBRT patients immobilized in the BodyFix system. Patient rotations due to intrafraction motion and between IMRT and VMAT were not clinically significant due to the majority of the measurements falling below the correction threshold of 1°. However, despite rotational setup error remaining clinically insignificant with regard to intrafraction motion, it remains an important factor at initial patient setup where complex breathing motion reduces the accuracy of tattoo alignment to external markers and resulted in 84% and 43% of patients falling above the correction threshold of 1° and 2°, respectively; no patients exhibited a rotational error > 3°. The importance of correcting both translational and rotational setup error is highlighted in the use of steep dose gradients, such as in SBRT, where misalignments can more readily result in a decrease in target coverage and increase in OAR doses.^(14)^ Overall, the 95% confidence interval of 2.1 mm and 0.8° demonstrates that the isotropic PTV margin of 5 mm is safe.

Breathing‐induced kidney and target intrafraction motion is greatest in the superior–inferior direction in patients positioned in the dual‐vacuum‐cushion immobilization system. In this system, an ITV contoured from the inhale, exhale, and average phases of the 4D CT dataset with a 5 mm isotropic PTV margin is safe to account for target volume intrafraction motion and patient setup accuracy. Evaluation of translational and rotational patient setup errors during highly conformal hypofractionated treatments is necessary to ensure accurate localization of highly mobile targets. Correction of both translational and rotational misalignments is necessary to achieve the improved dose delivery accuracy required of SBRT. Alternate motion management and treatment delivery strategies may further reduce kidney motion, reduce setup errors, and improve target localization.

## COPYRIGHT

This work is licensed under a Creative Commons Attribution 3.0 Unported License.
